# Mechanisms and Clinical Significance of Tumor Lymphatic Invasion

**DOI:** 10.3390/cells10102585

**Published:** 2021-09-29

**Authors:** Noriki Fujimoto, Lothar C. Dieterich

**Affiliations:** 1 Department of Dermatology, Shiga University of Medical Science, Otsu 520-2192, Japan; noriki@belle.shiga-med.ac.jp; 2 Institute of Pharmaceutical Sciences, Swiss Federal Institute of Technology (ETH) Zurich, Vladimir-Prelog-Weg 1-5/10, 8093 Zurich, Switzerland

**Keywords:** tumor lymphangiogenesis, lymphatic invasion, metastasis, lymph node, lymphatic endothelial cell

## Abstract

Tumor-associated lymphatic vessels play an important role in tumor progression, mediating lymphatic dissemination of malignant cells to tumor-draining lymph nodes and regulating tumor immunity. An early, necessary step in the lymphatic metastasis cascade is the invasion of lymphatic vessels by tumor cell clusters or single tumor cells. In this review, we discuss our current understanding of the underlying cellular and molecular mechanisms, which include tumor-specific as well as normal, developmental and immunological processes “hijacked” by tumor cells to gain access to the lymphatic system. Furthermore, we summarize the prognostic value of lymphatic invasion, discuss its relationship with local recurrence, lymph node and distant metastasis, and highlight potential therapeutic options and challenges.

## 1. Introduction

**Figure 1:**
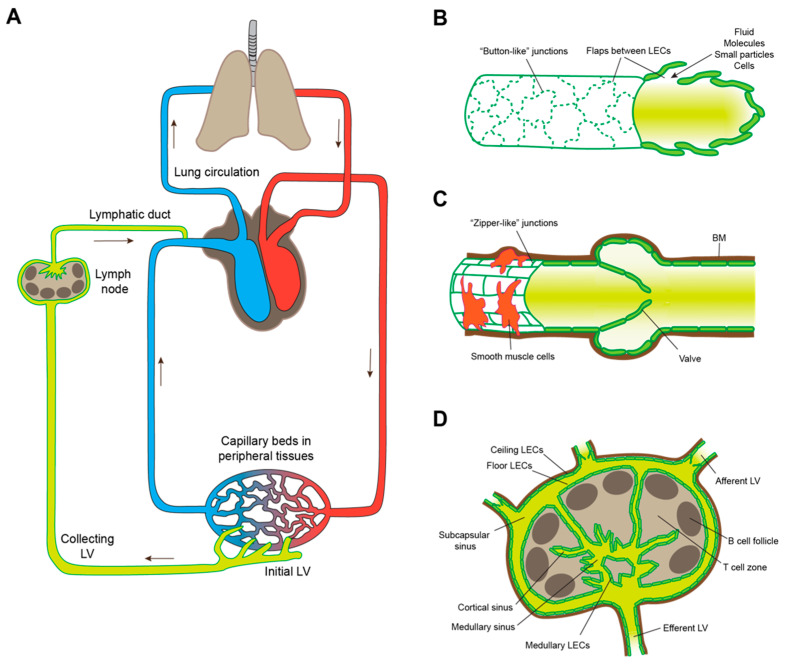
Overview of the lymphatic system. (**A**) Integrated schematic representation of the blood circulation and the lymphatic system. LV: lymphatic vessel. (**B**) Anatomical characteristics of initial lymphatic vessels. The individual LECs are irregularly shaped and partly overlapping. Junctions between LECs are discontinuous (“button-like”), facilitating intravasation of fluid, molecules, particles, and entire cells from the interstitium via LEC flaps. (**C**) Anatomical characteristics of collecting lymphatic vessels. The LECs are regularly shaped and form tight junctions (“zipper-like”) between them. Perivascular smooth muscle cells support the vessel and allow it to contract. Furthermore, collecting lymphatic vessels have a continuous basement membrane (BM) and valves to ensure unidirectional lymph transport. (**D**) Schematic representation of a lymph node with afferent and efferent lymphatic vessels, various types of sinuses, and corresponding LEC subsets.

The lymphatic system is a blind-ended drainage system, composed of initial lymphatic networks present in almost every organ, collecting vessels and lymph nodes (LNs). Its physiological functions include drainage of interstitial spaces in peripheral tissues to maintain fluid homeostasis, transport of antigen, antigen-presenting cells and lymphocytes from the body periphery to LNs and from LNs to the systemic circulation, as well as lipid transport [1,2]. Lymphatic uptake of interstitial fluid and soluble molecules, but also of larger molecular aggregates, particles and entire cells, occurs at the level of the blind-ended initial lymphatic vessels. From there, lymph is transported through afferent collecting vessels to draining LNs, percolates through a network of lymphatic sinuses, and then leaves the LN via a single efferent collecting vessel that connects to secondary LNs and ultimately to the venous circulation via the thoracic duct (left lymphatic duct) or the right lymphatic duct (Figure 1A). Initial and collecting lymphatic vessels bear remarkable anatomical adaptations to fulfill their respective tasks. Likewise, lymphatic endothelial cells (LECs) that line all of these vessels as well as lymphatic sinuses within LNs are heterogenous and display various molecular phenotypes depending on the type of the lymphatic bed and the organ in which they are located [3]. Initial lymphatics are thin-walled vessels, largely lacking perivascular supporting cells, and with a discontinuous basement membrane, facilitating cellular entry. Another remarkable feature of those vessels is the irregular (“oak-leaf”) shape of the LECs lining them, and the “button-like” junctions between adjacent LECs, in which patches of tight connections stabilized by VE-cadherin are interspersed with large flaps of partly overlapping LECs that allow for easy intravasation of tissue-emigrating leukocytes, such as dendritic cells (DCs) (Figure 1B). Furthermore, LECs of initial lymphatics constitutively express the chemokine CCL21 that guides DCs and other tissue-emigrating cells towards them. In contrast, collecting lymphatic vessels are supported by smooth muscle cells that allow the vessels to contract and thereby pump the lymph forward, even against pressure gradients. Furthermore, LECs of collecting vessels are smooth and elongated in shape, and form continuous, tight, “zipper-like” junctions. In addition, collecting lymphatic vessels have valves that ensure unidirectional lymph transport (Figure 1C). The lymphovenous valve, located at the junction between the lymphatic ducts and the subclavian veins, prevents blood entry to the lymphatic system. Finally, there are several distinct but continuous lymphatic sinuses within LNs (Figure 1D). Afferent lymphatic vessels discharge into the subcapsular sinus surrounding almost the entire LN parenchyma. This sinus directly connects to a network of cortical and medullary sinuses, which link to the efferent lymphatic vessel. Interestingly, recent single-cell RNA sequencing studies have shown that LN-resident LECs lining those sinuses are phenotypically and functionally heterogenous [4,5,6]. LECs of the outer wall (“ceiling LECs”) of the subcapsular sinus share phenotypic characteristics with LECs of collecting lymphatic vessels and presumably contribute to a tight barrier towards the surrounding tissue, whereas those of the inner lining of the subcapsular sinus (“floor LECs”) and those of cortical and medullary sinuses are more reminiscent of initial LECs and are permissive for transendothelial transport and migration of leukocytes between the lymphatic lumen and the LN parenchyma [7].

Due to their function as fluid drainage and molecular and cellular transport system, lymphatic vessels show dynamic phenotypic responses and play important roles in a wide range of pathological conditions, including acute and chronic inflammation as well as cancer [8,9,10]. It has been known for a long time that solid tumor growth not only induces the formation of new blood vessels (angiogenesis), but in some cases also of lymphatic vessels (lymphangiogenesis). This is mediated by various lymphangiogenic factors that are released by tumor cells or host-derived cells (immune cells, fibroblasts, etc.) present within the tumor microenvironment (TME). These include vascular endothelial growth factor C (VEGF-C) and D, which bind to and stimulate their receptor VEGFR-3 expressed by LECs; VEGF-A, which stimulates both angiogenesis and lymphangiogenesis by binding to VEGFR-2; angiopoietin-2; hepatocyte growth factor (HGF); fibroblast growth factor (FGF); etc. (reviewed in [8]). Similarly, LECs in tumor-draining LNs have been shown to proliferate, leading to the expansion of nodal sinuses [11], whereas tumor-draining collecting vessels dilate and show increased pumping frequency [12,13], leading to enhanced lymph transport.

Importantly, accumulating clinical data demonstrates that the density of lymphatic vessels within or surrounding growing tumors correlates with poor patient outcome. This applies to many types of solid tumors, even those that do not prominently induce lymphangiogenesis, suggesting that the lymphatic system contributes to tumor progression [8,10,14]. Furthermore, at least in melanoma, the same correlation can be observed at metastatic sites in the lung [15]. Clearly, tumor-associated lymphatic vessels are involved in malignant dissemination, since the first site of overt metastasis is very often the tumor-draining LN. Additionally, the lymphatic system has been shown to play an important role in tumor immunity, on the one hand transporting tumor antigens to draining LNs which is necessary for the activation of T cell responses [16,17], while on the other hand directly inhibiting tumor-specific T cell responses by LEC expression of immune checkpoint molecules, such as PD-L1 [18,19,20].

In this review, we focus on an early, essential step in the metastatic dissemination of malignant tumor cells, namely their entry into lymphatic vessels, a process called lymphatic vascular invasion (LVI). We discuss the current knowledge about molecular and cellular mechanisms mediating this process, its clinical significance, and conceptual and technical challenges for therapeutic intervention with LVI.

## 2. Lymphatic Intravasation Processes in the Healthy Organism

### 2.1. Lymphatic Entry by Leukocytes

**Figure 2:**
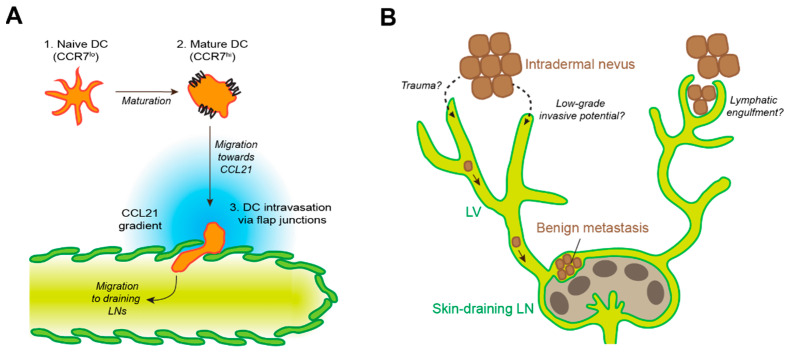
Lymphatic intravasation in the healthy organism. (**A**) Schematic representation of DC entry into initial lymphatic vessels. Upon maturation, DCs upregulate expression of CCR7, a receptor for the LEC-expressed chemokine CCL21, enabling them to navigate towards a nearby lymphatic vessel and to enter the lumen via flap junctions between adjacent LECs. In inflammatory conditions, additional chemokines and adhesion molecules may be involved in this process. (**B**) Illustration of the benign metastasis process. Occasionally, benign stromal cells, e.g., dermal nevus cells, can enter lymphatic vessels (LVs) and reach draining lymph nodes, where they form clusters within the subcapsular sinus. Lymphatic entry may be mediated by minor trauma, low-grade invasive potential of the nevus cells, or by lymphatic engulfment of single or clustered nevus cells.

As outlined above, initial lymphatic vessels in peripheral tissues bear multiple molecular and micro-anatomical features that greatly facilitate the passage of other cells, in particular of leukocytes, from the interstitium into the lymphatic lumen. Clearly, leukocyte lymphatic intravasation and re-circulation from peripheral tissues via LNs to the systemic circulation is essential for immune surveillance, and the underlying molecular mechanisms have been studied to considerable detail, in particular in the case of DCs (reviewed in [21,22], Figure 2A). DCs are a heterogenous cell population essential for adaptive immunity and can be found in virtually all peripheral tissues [23]. Resting DCs are typically poorly motile but have a high phagocytic activity, enabling them to sample antigen from their surroundings. In contrast, if activated by appropriate signals such as inflammatory mediators, pathogen- or danger-associated molecular patterns, DCs mature, which increases their capacity to prime or activate lymphocytes by upregulation of their antigen-presentation and co-stimulatory machinery and reduction of their phagocytic activity. In addition, DC maturation provokes upregulation of chemokine receptors, most importantly of CCR7, and increases DC motility. Thus, mature DCs are able to move through the surrounding tissue in an ameboid fashion, forming protrusions and squeezing through the tight interstitial spaces, and to sense gradients of the chemokine CCL21 that guide them towards the nearest lymphatic vessel [24,25,26]. For lymphatic intravasation, DCs again squeeze through the sparse basement membrane and the endothelial flap junctions of initial lymphatics. Consequently, at least under steady-state conditions, the entire process of DC migration into lymphatics is strongly dependent on CCL21 –CCR7 signaling [27,28,29] but is largely independent of integrins and of endothelial adhesion molecules that are involved in leukocyte extravasation from blood vessels. Inflammatory conditions induce the release of additional DC guidance cues from LECs, including chemokines such as CXCL12 and sphingosine-1-phosphate (S1P), which contribute to DC migration towards lymphatic vessels [30,31,32,33]. Furthermore, LECs upregulate various leukocyte adhesion molecules such as ICAM-1 and VCAM-1, which facilitate DC intravasation into initial lymphatics [33,34]. Interestingly, VCAM-1 was recently shown to enable DC intravasation even into collecting lymphatic vessels that are typically not permissive for cellular entry [35]. Next to DCs, T cells represent another prominent type of leukocytes that are able to migrate from peripheral tissues to draining LNs via afferent lymphatic vessels. In fact, T cells represent the majority of lymphatic leukocytes under steady-state conditions. Similar to DCs, T cells strongly depend on CCR7/CCL21 as well as S1P signaling during tissue egress [36,37,38]. Furthermore, S1P orchestrates lymphocyte egress from LNs directing them from the LN parenchyma into lymphatic sinuses and efferent lymphatics [39]. Finally, several adhesion molecules expressed by LECs, including MRC-1 and CLEVER-1, have been reported to mediate lymphatic intravasation of T cells in peripheral tissues (reviewed in [21,22]).

### 2.2. Benign Metastasis

There is evidence that, occasionally, LVI may occur not only in malignant tumors, but also in benign lesions such as melanocytic nevus, blue nevus, Spitz nevus, clear cell hidradenoma, uterine leiomyoma, etc. [40,41,42,43,44]. This phenomenon is often referred to as “benign metastasis” (Figure 2B). McCarthy reported that benign nevus cells were found in LNs from 24 patients out of a cohort of 362 patients [40]. In these 24 patients, nevus cells were always located in the subcapsular sinus, often in trabecular sinuses, and rarely within the LN parenchyma. Two possibilities have been discussed regarding the origin of benign nevus cells in LNs [45]. First, these cells may be the result of an anomaly of histogenesis. This possibility is unlikely because nevus cells have never been found in deep LNs such as abdominal, thoracic or iliac nodes but exclusively in superficial LNs such as in the axilla, neck, and groin regions [40]. The second possibility is a migration of these cells from a nevus in the skin to LNs. This possibility, the so-called mechanical transport theory, is supported by several observations: (A) among the above 24 patients in whom nevus cells were found in LNs, nevus cells were located in the subcapsular sinus in all patients and benign nevi were found in the skin drained by these LNs in 21 patients [40]; (B) intralymphatic nevus cell aggregates are sometimes found in the dermis in excisional biopsy samples of benign cutaneous nevi [46]; (C) benign tissues such as thyroid or salivary gland are infrequently found in draining LNs from patients with head and neck carcinoma or adenocarcinoma of the lung [47,48]. While the mechanism by which nevus cells migrate form the skin to draining LNs through lymphatic vessels is unclear, two major hypotheses have been brought forward. On the one hand, nevus cells might enter lymphatic vessels by themselves. On the other hand, lymphatic vessels might “integrate” or engulf nevus cells. In some cases, such as Spitz nevus and clear cell hidradenoma, the benign tumor cells may have some malignant and/or invasive potential [42,43], enabling them to invade lymphatic vessels and to migrate to draining LNs, perhaps employing mechanisms similar to those discussed below (Section 4). Indeed, molecular analysis from eruptive Spitz nevi demonstrated the presence of a ROS1 fusion oncogene, which may be associated with benign metastasis [42]. As for cases with clear cell hidradenoma, histopathologic findings demonstrated benign features and long-term follow-up revealed a benign prognosis, but the tumor cells appeared to form much larger nests in the LNs compared to nevus cells [43]. Although genetic analysis has not been performed, these cells may have uncertain low-grade malignant potential. In cases of benign cutaneous nevi, it has been suggested that local minor trauma may cause intralymphatic nevus cell aggregates, which eventually transfer nevus cell to LNs [46]. A deeper understanding of the molecular mechanisms underlying this interesting phenomenon of benign metastasis could provide new insights into the pathogenesis of LVI of malignant tumor cells.

## 3. Local Invasion

**Figure 3:**
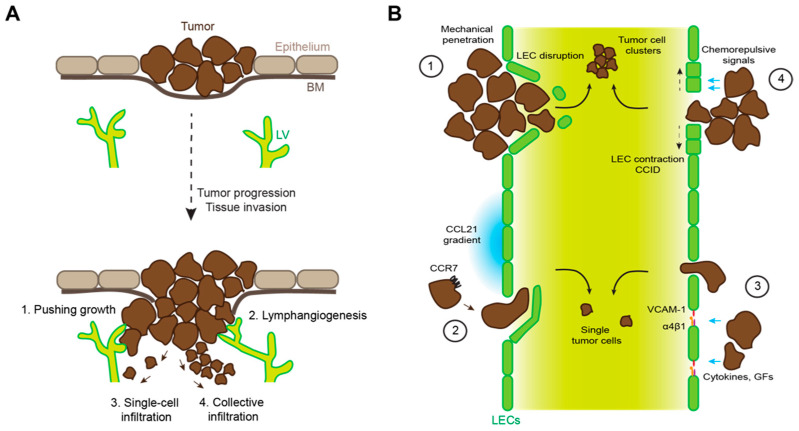
Mechanisms of tumor lymphatic invasion. (**A**) Schematic representation of progression from an in situ to an invasive carcinoma, breaching the basement membrane (BM) and growing into the underlying tissue where lymphatic vessels (LV) are located. Tumor cells can get access to lymphatic vessels by pushing growth of the tumor mass; induction of lymphangiogenesis in or around the tumor; single-cell tissue infiltration; and collective tissue infiltration. (**B**) Tumor cells can invade lymphatic vessels by (1) mechanical damage or disruption of the lymphatic endothelial wall; (2) by sensing CCL21 gradients and intravasation via lymphatic endothelial flap junctions; (3) by inducing lymphatic permeability, e.g., through cytokine- or growth factor (GF)-induced upregulation of α4β1 integrin and its ligand VCAM-1 in LECs; (4) via release of chemorepulsive agents (e.g., 12(S)-HETE) that induce LEC retraction and formation of invasion portals (CCIDs).

To metastasize, tumor cells have to gain access to the lymphatic or blood vasculature (Figure 3A). In the case of most cancer types (e.g., carcinomas and melanoma), this requires breaching of an epithelial basement membrane, followed by invasive growth into the underlying, vascularized tissue. Tumor–vascular interfaces may be created via “co-option” of pre-existing vessels, or by induction of peri- or intratumoral angiogenesis and lymphangiogenesis. In addition, tumor cells at the invasive front often exhibit an infiltrative behavior, penetrating into the surrounding tissue either as cell streaks or clusters (collective infiltration) or as single cells. In carcinomas, this is strongly dependent on “epithelial-to-mesenchymal transition” (EMT), an evolutionary conserved cellular program essential for embryogenesis and pathological responses such as wound healing or tissue repair [49,50]. In the tumor context, EMT is induced by various signals from the tumor microenvironment, including TGF-β or Wnt signaling, inflammation, and hypoxia, which induce the expression of several key transcription factors of the Snail, Twist, and Zeb families [51,52]. In turn, these transcription factors mediate massive phenotypic changes in carcinoma cells, downregulating epithelial traits (including cell–cell junctions and cell polarity) while inducing mesenchymal characteristics such as cytoskeletal remodeling and expression of ECM-degrading proteases, enabling the cells to invade the surrounding tissue. It is important to note that EMT is a gradual process. Complete EMT is probably a rare event in cancer progression. Instead, infiltrating tumor cells have been described to undergo “partial” or “hybrid” EMT, leading to a mixed phenotype with both epithelial and mesenchymal traits, favoring collective over single-cell infiltration [53,54]. Indeed, tumor cells in patients suffering from various types of cancer have been reported to infiltrate collectively, whereas single infiltrating cells were very rare [55]. Furthermore, vessel-invading tumor cells frequently present as cell clusters or emboli, and tumor cells at metastatic sites express typical epithelial genes, arguing against a major role of complete EMT during tissue invasion [52,56].

With regard to tumor cell EMT, melanoma represents a special case. Melanocytes are derived from embryonic progenitor cells in the neural crest that undergo EMT in order to migrate through the embryo and colonize the epidermis [57]. During this process, the cells differentiate into mature melanocytes that acquire E-cadherin expression [58], but are not considered as epithelial cells. It is tempting to speculate that this hybrid phenotype and “mesenchymal heritage” of melanocytes is related to their propensity to form benign metastasis as discussed above. Nonetheless, “EMT-like” dedifferentiation of melanoma cells has been shown to contribute to tumor cell invasion, much like EMT in carcinomas [59]. In addition, melanoma collective migration and LVI was shown to be regulated by tumor cell expression of the cell surface protease MMP16, which cleaved and de-activated molecules involved in single cell migration and blood vessel invasion [60].

Another important factor influencing the migratory behavior of tissue-infiltrating cancer cells is the extracellular matrix (ECM) which is often abnormal and/or undergoing constant remodeling within the tumor mass and the tumor periphery due to the activity of various cell types present within the TME, such as cancer-associated fibroblasts (CAFs), tumor-associated macrophages (TAMs), and cancer cells themselves. For an in-depth discussion of this topic, we refer the reader to several comprehensive, recent review articles [61,62,63].

## 4. Mechanisms of Tumor Lymphatic Invasion

### 4.1. Mechanical Disruption of Endothelial Barriers

Once in contact with the vasculature, the next step in the metastatic cascade is vascular invasion by penetrating the perivascular area, basement membrane, and finally, the endothelial layer. Various specific molecular and cellular mechanisms have been described that actively facilitate this process, many of which evolved to exert other functions, for instance in ontogeny, steady-state or pathological conditions (Table 1, Figure 3B). Yet, at least in the case of vessels within or very close to a rapidly growing tumor mass, LVI might be a “passive” process in which the uncontrolled expansion of tumor cells disturbs the normal cellular and matrix architecture of the surrounding tissue and exerts mechanical stress, including pressure and stretching forces, on neighboring cells. The delicate initial lymphatic vessels that lack a continuous basement membrane and support by perivascular cells may be particularly sensitive to such physical stress. Indeed, histological studies in human cancer tissue have suggested that clusters of tumor cells mechanically penetrate lymphatic and blood vessels and destroy the endothelium, although it is difficult to proof experimentally that this is really due to mechanical stress [64,65].

### 4.2. Immune Cell Mimicry

Tumor cells frequently dysregulate and exploit normal cellular processes that exert important functions during embryogenesis or responses to pathological conditions, such as the above discussed EMT. Another example is LVI by tumor cells “hijacking” signaling cues for leukocyte intravasation. For example, pre-clinical studies have demonstrated that CCR7 expression in tumor cells promotes migration towards lymphatic vessels, LVI, and LN metastasis [66,67,68,69,70]. Congruently, in clinical studies CCR7 expression correlated with LVI, LN metastasis and poor outcome in various cancer types such as gastric cancer, pancreatic cancer, non-small cell lung cancer, colorectal carcinoma, and cervical cancer [68,70,71,72,73]. Interestingly, CCR7 signaling has also been reported to be involved in the amplification of a pool of cancer stem-like cells in breast cancer [74]. Furthermore, TGF-β-induced EMT has been identified as a trigger of CCR7 expression in breast cancer cells [75].

Next to the CCL21–CCR7 axis, other chemokine receptors, such as CXCR4 sensing lymphatic CXCL12, have been reported to guide tumor cells towards and into lymphatic vessels and to promote LN metastasis [66,76,77]. Additionally, tumor-associated LECs have been shown to upregulate CXCL1 that induced LVI in gastric cancer [78]. However, as the role of CXCL1 in immune cell intravasation of lymphatic vessels is not clear, this case may not reflect immune cell mimicry by tumor cells.

While the role of chemokines in tumor LVI is well established, the function of lymphatic endothelial adhesion molecules is unclear. VCAM-1 expression mediates adhesion of melanoma cells to LECs in vitro, but this was not the case for breast cancer cells [79,80], and there is no animal or clinical data supporting a role of VCAM-1 in tumor cell-LEC adhesion in vivo. On the other hand, lymphatic endothelial expression of CLEVER-1 and ESAM were found to correlated with LN metastasis in breast cancer, head-and-neck cancer, and colorectal carcinoma, but it is not known whether this involved increased LVI and/or adhesion between tumor cells and LECs [81,82].

Hematological malignancies that colonize LNs such as chronic lymphatic leukemia (CLL) represent a special case of immune cell mimicry by tumor cells entering lymphatic vessels. CLL cells transmigrate from the blood stream into the LN parenchyma via high endothelial venules in a CCR7-dependent manner (reviewed in [83]). There, CLL cells accumulate due to proliferation and impaired LN egress. Nonetheless, CLL cells do express low levels of the S1P receptor 1 (S1PR1), enabling them to enter lymphatic sinuses and reach efferent lymphatic vessels essentially in the same way as lymphocytes egress from LNs. Of note, S1PR1 expression and lymphatic migration of CLL cells is induced by drugs such as idelalisib [84].

### 4.3. Tumor-Induced Destabilization of Lymphatic Junctions

Although the cell–cell junctions of initial lymphatic vessels are discontinuous and adapted for leukocyte intravasation, the gaps between VE-cadherin-positive “buttons” are very small (ca. 3 µm in mouse trachea [85]). Thus, lymphatic junctions in both initials and collecting lymphatic vessels represent a barrier to tumor cells that are on average larger and less deformable than leukocytes [86,87]. However, signals derived from tumor cells or the TME can impair the integrity of lymphatic endothelial junctions and increase vessel permeability, which may facilitate LVI. For instance, in breast cancer, a combination of inflammatory signals and growth factors induces lymphatic expression of both VCAM-1 and its receptor α4β1 integrin, leading to the reduction of junctional VE-cadherin [79,88], similar to the remodeling of blood endothelial junctions during leukocyte extravasation [89]. In addition, lymphatic VE-cadherin expression is reduced by chronic lymphangiogenic signaling via VEGF-C in colorectal carcinoma [90] and by tumor-cell-derived serum amyloid A in a breast cancer model [91]. Finally, a distinct mechanism involving low-molecular-weight hyaluronan binding to lymphatic LYVE-1 and subsequent destabilization of tight junctions has been described in vitro [92], but its relevance for LVI in vivo is unknown.

### 4.4. Tumor-Induced Formation of Entry Portals into Lymphatic Vessels

A less subtle mechanism of tumor LVI than junctional remodeling is the formation or large holes in the endothelial lining, which allow entire tumor cell clusters to reach the lymphatic lumen. Such holes can be the result of tumor-cell-derived signals that are chemorepulsive for LECs and have therefore been denoted as “chemorepellent-induced defects” (CCIDs) or entry gates. Using an in vitro model of breast cancer LVI, it was shown that CCID formation was induced by the enzyme arachidonate 15-lipoxygenase (Alox15), which catalyzes the generation of various bioactive lipid mediators including 12(S)-hydroxyeicosatetraenoic acid (12(S)-HETE) that is chemorepulsive (but not toxic) for LECs [93]. Consequently, Alox15 depletion resulted in reduced LN metastasis in a xenograft model of breast cancer, and Alox15 expression in LN metastasis correlated with secondary metastasis in breast cancer patients [93]. Of note, the same mechanism has been shown to mediate lymphatic intravasation of neutrophils in inflammatory conditions [94]. Subsequently, lymphatic CCID formation was also shown using pancreatic cancer cells, although the underlying molecular mechanism differed in this case [95].

### 4.5. Role of Bystander Cells in Lymphatic Invasion

Local tissue invasion and LVI by tumor cells may be supported by cells in the TME, for instance immune cells and fibroblasts. This is particularly well documented for TAMs. Although a heterogenous population, TAMs often assume a phenotype associated with wound healing and tissue repair, which impairs tumor immunity and promotes (lymph-) angiogenesis. Furthermore, TAMs express multiple proteases and are able to degrade or remodel the ECM, which facilitates tumor cell migration in the tissue [96]. In breast cancer, TAMs have also been shown to aid the actual step of LVI. For instance, two studies found that macrophages positioned close to lymphatic vessels via β4 integrin and/or interaction with lymphatic galectin-8 induced vessel dilation, permeability, matrix remodeling and invasion [97,98]. Similarly, tumor-infiltrating neutrophils as well as innate lymphoid type 3 cells also promote tissue invasion by tumor cells via release of ECM-degrading enzymes such as elastase, cathepsins, MMPs, or RANKL, respectively [99,100]. Another important cell type in the tumor stroma with invasion- and metastasis-promoting capacities are CAFs, mesenchymal cells that, similar to TAMs, are strongly involved in ECM generation and remodeling, regulation of (lymph-) angiogenesis, and the induction of EMT via release of proteases and ECM components, chemokines, TGF-β, and Wnt ligands (reviewed in [101,102]).

## 5. Clinical Implications of Lymphatic Invasion—From Prognosis to Therapy?

### 5.1. Prognostic Value of Tumor LVI

LVI of tumor cells is an early, necessary step for lymphatic metastasis. Traditionally, clinical evaluation of LVI has relied on hematoxylin/eosin (HE)-stained tissue sections. However, due to various technical reasons, this approach significantly underestimated the occurrence of LVI. Once antibodies to specifically detect lymphatic endothelium, such as anti-LYVE-1 antibodies detecting lymphatic initials or the D2-40 antibody specific for the pan-lymphatic marker podoplanin, were introduced into diagnostic practice, a much higher frequency of LVI could be detected [103]. Although the clinical data that have accumulated over the last couple of decades is not equally clear in every type of cancer (i.e., there have been debates about the prognostic value of LVI in melanoma and colorectal carcinoma), a large number of clinical studies using both HE- and antibody-mediated detection of LVI has clearly established its prognostic value in these and various other cancer types (Table 2). Consequently, a significant correlation between LVI, LN metastasis and/or poor outcome has also been detected in large meta-analysis studies including hundreds of patients with breast cancer, melanoma, and colorectal carcinoma [104,105,106,107].

### 5.2. What Is the Relationship between Lymphatic Metastasis and Distant Metastasis?

Given the strong correlation between tumor LVI, dissemination via lymphatic vessels and poor clinical outcome, therapeutic targeting of this process might appear as a meaningful approach to hinder tumor progression. The reality, however, is more complex. Although there is some evidence suggesting that LVI contributes to local (or locoregional) recurrence [129,147,148,149], perhaps by creating a reservoir or a lymphatic niche for long-term maintenance of tumor cells in proximity to the original tumor bed, there are very few data, either from pre-clinical or clinical research, consistent with a causative relationship between lymphatic and distant metastasis. In fact, two multicenter randomized-control trials (DECoG-SLT and MSLT-II) of melanoma patients with sentinel LN metastasis randomly assigned to complete LN dissection vs. watchful waiting revealed no difference in patient survival or distant metastasis [150,151]. On the contrary, LN dissection was associated with significant adverse effects, such as lymphedema. Similarly, breast cancer patients with sentinel LN metastasis experienced no benefit, neither in terms of regional recurrence-free survival nor overall survival, from complete axillary LN dissection as compared to sentinel LN dissection only [152]. Thus, it is unlikely that metastatic LNs represent a significant reservoir for distant dissemination, at least not in melanoma and breast cancer and at the timepoint of treatment. In fact, LNs could even be regarded as a metastasis barrier or “filter”, preventing tumor cells from spreading systemically and exposing them directly to cells of the adaptive immune system. Although tumor-draining LNs have traditionally been regarded as immunocompromised sites that are not conducive for efficient anti-tumor immune responses [153,154], recent data has shown LN-resident CD8^+^ T cells efficiently delete metastatic tumor cells in a mouse melanoma model [155].

On the other hand, metastatic cells within tumor-draining LNs clearly can seed to distant sites, either via node-associated blood vessels or efferent lymphatic vessels and the lymphatic ducts. This has been shown in cancer models in mice by injection of tumor cells directly into afferent lymphatic vessels and by use of a photoswitchable tumor cell model [156,157]. In cancer patients, mutational analysis allowed the reconstruction of genetic relationships and probable metastatic pathways between individual metastatic sites. For instance, in prostate cancer, complex patterns of organ-to-organ metastasis, sometimes involving tumor-draining LNs, have been mapped [158,159], whereas in colorectal carcinoma, up to a third of distant metastases were likely derived from LN metastasis [160,161]. In conclusion, in the majority of cases, LN metastasis appears to be an indicator of tumor aggressiveness and metastatic potential, while a direct causative relationship in individual cases cannot be excluded and might depend on the tumor (sub-)type or the timepoint of analysis.

### 5.3. Potential Therapeutic Approaches

Therapeutic intervention with LVI or lymphatic dissemination in general is challenging for several reasons. As discussed above, a better understanding of the relationship between lymphatic and distant metastasis in various types of cancer is needed to identify conditions in which such a treatment might be beneficial. Secondly, the timing of the treatment and its integration into the indicated cancer-directed therapies are challenging. For example, targeting of LVI or dissemination would be most impactful if administered at very early stages of tumor progression when lymphatic spreading has not yet occurred. However, cancer patients often present when their tumors have already progressed. Furthermore, the treatment is most meaningful in unresectable disease or accompanying neo-adjuvant therapy. Thirdly, care must be taken that there is no negative impact on the normal, essential functions of lymphatic vessels, including the transport of antigen-presenting cells, which is important for tumor immunity. Therefore, targeting of the CCL21–CCR7 axis might not be a valid approach.

**Figure 4:**
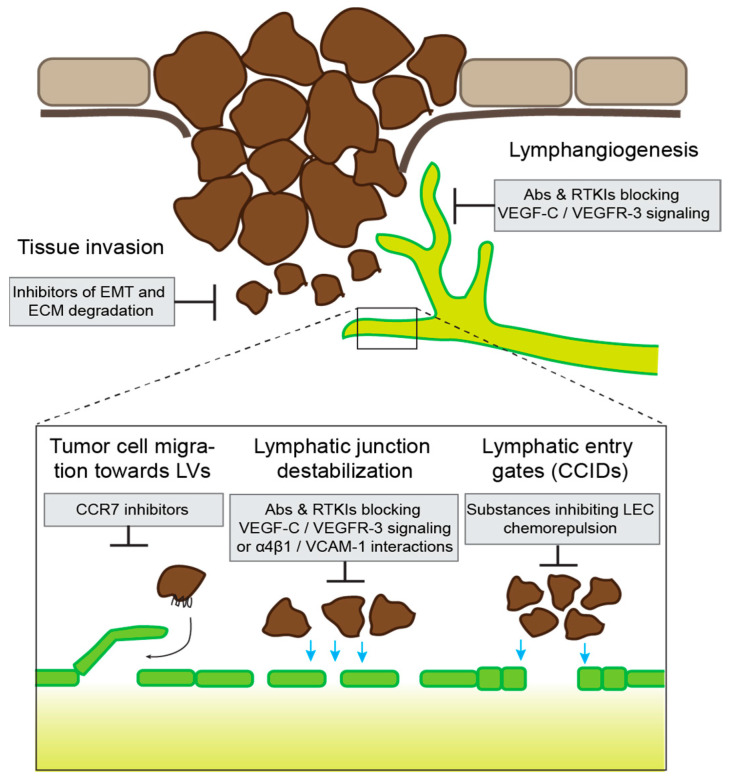
Potential therapeutic approaches to prevent lymphatic invasion. Overview of some of the main steps and mechanisms of tumor lymphatic invasion and potential inhibitory drugs. Abs: antibodies; RTKIs: receptor tyrosine kinase inhibitors; LVs: lymphatic vessels.

Despite these challenges, several drugs aiming to inhibit LVI and lymphatic dissemination are currently in pre-clinical or clinical development (Figure 4). The first step towards LVI is local tissue infiltration by tumor cells, and several approaches or therapeutics have been designed to interfere with this process, for instance blockade of EMT, inhibition of matrix-degrading enzymes, or depletion or reprogramming of TAMs (reviewed in [162,163,164]). However, many of these approaches have shown only limited efficacy, and/or concerns about significant adverse effects have been raised. Another strategy is the inhibition of tumor-associated lymphangiogenesis, resulting in a reduced tumor–lymphatic interface. This can be achieved by targeting major lymphangiogenic pathways, such as the VEGF-C–VEGFR-3 pathway, the angiopoietin pathway, or HGF–c-MET signaling [8]. Indeed, pre-clinical studies have shown that interference with these pathways can potently reduce lymphatic metastasis in cancer models in mice [165,166,167,168]. Although at least in part developed for other purposes, neutralizing antibodies against VEGF-C (VGX-100), its receptor VEGFR-3 (IMC-3C5), angiopoietin-2 (Nesvacumab) or HGF (Ficlatuzumab) have been or are currently undergoing clinical testing (Clinicaltrials.gov (accessed on 6 September 2021) NCT01514123, NCT01288989, NCT01271972, NCT00969410). In addition, there are multiple receptor tyrosine kinase inhibitors in clinical use or in testing that could impede tumor lymphangiogenesis based on their target profile [8]. However, additional studies are required to evaluate if any of those drugs could be purposed for the prevention of lymphatic metastasis. The third potential level for therapeutic intervention is the actual step of LVI. As outlined above, several specific molecular and cellular mechanisms have been identified that can facilitate the intravasation of tumor cells. For instance, in a breast cancer model in mice, blockade of VCAM-1 reduced tumor LVI, presumably due to its role in destabilizing lymphatic endothelial junctions by engaging α4β1 integrin on adjacent LECs [79]. Although there is currently no VCAM-1-blocking drug in clinical development, an antibody blocking α4β1 (Natalizumab) has already been approved for clinical use. A potential application of this antibody to improve lymphatic junctional integrity, at least in conditions where α4β1 expression is induced in LECs, has not been tested so far. Finally, in vitro drug screens have identified potential inhibitors of CCID formation, including several FDA-approved substances such as acetohexamide or ketotifen [169,170], but their potential anti-metastatic activity in vivo has not been investigated.

## 6. Conclusions and Open Questions

The prognostic value of tumor LVI has been recognized in various cancer types for decades. However, there are still many open questions remaining. Our current understanding of the molecular and cellular processes mediating LVI is largely based on in vitro and animal models, and their clinical relevance is unclear. Another highly important question to be resolved regards the relationship between lymphatic and distant metastasis. A much better understanding in which cancer types and to what extent lymphatic dissemination is causally linked to distant tumor spread, perhaps through a higher resolution genetic tracking of metastatic tumor cell clones, would be pivotal for the future development of drugs for therapeutic intervention with LVI.

## Figures and Tables

**Table 1:** Molecular and cellular processes involved in tumor lymphatic invasion in relation to other functions in ontogeny, steady-state or pathological conditions.

Process	Role in LVI	Other Functions
EMT	Increased invasiveness and tissue infiltration of tumor cells.	Essential for ontogeny, wound healing, etc.
LEC-derived chemokine gradients.	Guidance of disseminating tumor cells towards/into initial lymphatic vessels.	Guidance of DCs and other immune cells towards/into initial lymphatic vessels.
Destabilization of lymphatic endothelial junctions.	Promotion of tumor trans-endothelial migration into the lymphatic lumen.	Unknown. Potentially involved in the regulation of lymphatic transport in inflammatory conditions.
Formation of entry gates (CCIDs) by LEC-repulsive signals.	Promotion of tumor cell cluster entry into lymphatic vessels.	Lymphatic entry of neutrophils in inflammatory conditions.

**Table 2:** Clinical studies showing an association between lymphatic invasion and poor outcome (LN metastasis, cancer recurrence, survival).

Cancer Type	LN Metastasis	Recurrence	Survival
Breast cancer	Schoppmann et al. [108] Mohammed et al. [109] El Gohary et al. [110] Zhao et al. [111]	Schoppmann et al. [108] Mohammed et al. [109] El Gohary et al. [110] Van der Schaft et al. [112] ^1^	Schoppmann et al. [108] Mohammed et al. [109] El Gohary et al. [110] Mohammed et al. [113]
Colorectal cancer	Tateishi et al. [114] Akagi et al. [115] Nishida et al. [116] Lee et al. [117]	Betge et al. [118] Iida et al. [119] Tokodai et al. [120] Leijssen et al. [121]	De Leon et al. [122] Guerra et al. [123] Akagi et al. [115] Betge et al. [118]
Lung cancer	Takanami, [124] Adachi et al. [125]	Kwiatkowski et al. [126] Kato et al. [127] Al-Alao et al. [128] Matsuura et al. [129]	Hanagiri et al. [130] Nentwich et al. [131] Masuda et al. [132]
SCC ^2^ of head and neck	Zhao et al. [133] Chung et al. [134] Adel et al. [135]	Hori et al. [136]	Myers et al. [137] ^3^ Mochiki et al. [138] Adel et al. [135] Casai et al. [139]
Melanoma	Bertolli et al. [140] Donizy et al. [141] Moy et al. [142] Jung et al. [143]	Donizy et al. [141] Moy et al. [142] Xu et al. [144] Statius Muller et al. [145]	Donizy et al. [141] Xu et al. [144] Vuylsteke et al. [146]

^1^ In patients < 63 years. ^2^ SCC: Squamous cell carcinoma. ^3^ In young adults.
